# Pure Food for Texas

**Published:** 1906-11

**Authors:** Walter R. Whitman

**Affiliations:** Editor Holland’s; Dallas, Texas


### Correspondence.

### Pure Food for Texas.

DALLAS, TEXAS, September 24, 1906.

*To the Editor Texas Medical Journal:*

Had you been with me on the trips I have made over Texas and seen the extent of adulteration and some of the vile things that go into our food products; had you seen, day after day, week after week, and month after month, the results of the chemist's analyses, as I have done, I believe you would feel as strongly as I do about the need of pure food legislation in Texas and would join earnestly in the effort to bring it about.

Much that I have seen I have not written about and shall probably not give to the public for two reasons. First, the Texas libel law throws some restrictions about the Texas newspaper man; and, second, some of the things were beyond belief unless a man actually saw them with his own eyes, and I do not care to have my own veracity or that of *Holland's* questioned.

The articles now appearing in *Holland's* are purposely stripped of word-painting and sensationalism so far as may be reasonably done; I am trying to give the public the plain, unvarnished truth as shown from scientific analyses, letting the people draw their own conclusions.

I most sincerely believe that the people of Texas should have an effective pure food law, and any man who will give one-hundredth part of the time I have given to food investigation will, I think, reach the same conclusion.

The papers of Texas can bring about the passage of the right kind of a law. Will you join in the fight? I would like to have you write me personally in regard to the matter.

Fraternally yours,

WALTER R. WHITMAN,

Editor *Holland's*.

That the "Red Back" will co-operate, goes without saying. D.

---

$2500-$3000.—Unopposed country practice free to purchaser of fine residence, office, outbuildings and three acres of good land; 100 miles west of Houston. Collections, 98 per cent; $2000, one-half cash, balance to suit purchaser. Address, "Doctor," box 28, Route 3, La Grange, Texas.

